# One leg at a time: medical librarians and fake news

**DOI:** 10.5195/jmla.2024.1858

**Published:** 2024-01-16

**Authors:** Michelle Kraft

**Affiliations:** 1 kraftm@ccf.org, Co-Lead Editor, Journal of the Medical Library Association, Medical Library Director, Cleveland Clinic Foundation, Cleveland, OH

## Abstract

While there has been recent media attention to the issue of “fake news,” misinformation and disinformation has been a lasting part of human history. This Janet Doe Lecture presents the history of fake news, how it is spread and accepted, its impact on medical and health information, and medical librarian roles in limiting its spread and promoting correct health information.

## INTRODUCTION

There is a saying, “A lie gets halfway around the world before the truth has a chance to get its pants on.” Jonathan Swift, Mark Twain, Winston Churchill, Terry Pratchett, and several famous people in history have been attributed to making this statement. It is difficult to determine who the originator of this statement was because it has been printed and reprinted many different times throughout history. However, historians agree that it is highly unlikely Churchill made such a statement which is a bit ironic given that it about the spread of a falsehood [1].

Fake news has been around probably since humans could write. Many people think fake news in politics is a recent issue but it has been used throughout history [2]. In 1769, John Adams wrote in his diary about spending the evening “Cooking up paragraphs, articles, occurrences, - working the political engine,” planting false and exaggerated stories to support the American revolution while undermining British rule [3]. In order to keep Germany in the dark about the Allies' new technology, RADAR, which enabled them to intercept and shoot down enemy bombers at night, the British government issued press releases stating that British pilots ate a lot of carrots to give them exceptional night vision. The disinformation campaign intended to fool Germans, but the British public seemed to believe the press releases as well leading to citizen gardens and 100,000-ton surplus of carrots in the country [2].

Not exclusive to governments and politics, there are many types of lies used for many types of reasons by many different people and groups. In her book, Killer Underwear Invasion! Elise Gravel reasons that fake news can be used to make money, be famous, spread beliefs or ideas, gain power, or share information often via social media [3]. Often times motives for spreading fake news is layered and involves several reasons at the same time.

Just as there are various types of fake news and reasons for it, there are several reasons why people are susceptible to fake news. A person's self-image, environment, and emotions all play a part in whether they believe and spread fake news.

When there aren't major clues about the fakeness of news items people will check its validity with other bits of knowledge to assess its compatibility. Information is more likely to be accepted as true by people when it is consistent with what they perceive or assume is true. If a message doesn't jive with one's own personal knowledge or beliefs, the message then gets stuck while trying to be processed, which causes resistance to the acceptance of the information without further investigation. In short, people endorse or support information that matches their preexisting beliefs or comes from an ideologically aligned source. It is easier to believe incorrect information than it is for a person to question their belief system.

People tend to associate and socialize with those who are familiar and have similar interests and social situations or environment. For example, people with children (the situation) might share information on the best vacation spots for kids, kid-friendly events, etc. By sharing this information with others in the same situation, they are sharing information that is familiar. These situations create bubbles that people live within further reinforcing our self-image and view within the bubble, which is their worldview. I use the term bubble to represent this type of worldview because a person's view within bubble is different than what it is when they are looking at something outside through the curvature and sheen of the bubble. The information within the bubbles remains within, bouncing around, reinforcing our beliefs internally and within the bubble.

Media fractionalization, the splitting of information among multiple media outlets through the creation and growth of cable TV, blogs, radio, internet, and social media have made it easier for people to find and select media information that already supports their existing worldview, reinforcing their self-image and bubble. People's online social activity would be considered cyber bubbles. Natalie Jomini Stroud in the Journal of Communication identified these cyber-bubbles as one reason for the increasing polarization of political discourse [4].

Recent studies have linked the relationship between emotions and susceptibility to fake news. Both positive and negative emotions appeared to selectively affect fake news judgement, while it had no impact on the belief in real news [5]. Skepticism was the only “emotion” not to affect people's judgement of the news. Interestingly, there was no meaningful correlation between emotions and political affiliation regarding believing fake news, emotions trumped the influence of the political party social bubble. The more people relied on emotion over reason, the more they perceived fake news to be accurate.

All of these things: self-image, environment, and emotions factor into our susceptibility to fake news. Stopping the spread of fake news is difficult, yet it is important. Nicole Cook states fake news is a “serious threat to information ecosystems, as truth is no longer related to authority, expertise, or real facts, but to interpretation, perception, emotions, and sentiments” [6]. Moreover, fake news is a symptom of greater problems as it politicizes and weaponizes information. The weaponization of information is against everything that librarians stand for. As librarians, we must find a way to stop the spread of fake news while promoting legitimate news. Stopping the spread is difficult, yet important. There are three ways to help stop the spread of fake news: debunking, debiasing, prebunking.

Debunking is not easy because it relies on changing a person's mind once they have already received the misinformation. In some situations, this can be effective and to have the best chance one must retract the misinformation with facts, repeat the correct information, and use credible sources. Despite doing these things, it can be extremely difficult because presenting the correct information that is contrary to the accepted fake news causes people to question a part of their sense of self, community, and their being. We see this very strongly with the anti-vax movement. They are a whole community where their world view includes the dangers of vaccines. Authors studying misinformation and its correction backfiring, determined when people opposed to vaccines were confronted with their benefits it increased their resistance to legitimate information on vaccines even more. The authors determined that exposure to belief-threatening evidence lead people to discount the entire scientific method. People would rather believe the issue cannot be solved scientifically rather than discounting information that goes against their beliefs [7].

Debiasing is talking to people and getting people to reject the fake news by changing their biases. It is similar to conversational debunking and has been likened to a dance where the fake news believer does much of the leading.

The person trying to do the debiasing must find the believer's worldview, use phrases that don't criticize their worldview but supports the factual parts of it, asks questions, and finds supportive peers within the fake news believer's worldview who trust the real news [7]. Debiasing typically does not happen in one conversation or with those who haven't built up a relationship. However, debiasing skills may be something that doctors, nurses, and other primary care providers who have more personal and continued relationships with patients can develop this type of communication method more.

Prebunking is the process of debunking the fake news and its sources before it happens. The idea behind prebunking is also called inoculation theory. A small amount of the virus, in this case fake news, can help people's minds ward off future exposure. A Cambridge research team created the “Bad News” game where the player's goal was to become a “disinformation and fake news tycoon” [8]. It was determined people playing the “Bad News” game were better at recognizing fake news than a control group playing a different game. Laura Garcia and Tommy Shane created a primer on the First Draft News website on prebunking tools, games, and recommendations [9].

Just like the intricate web of how people fall for fake news, the methods by which to expose misinformation and change people's belief in fake news is just as intricate and requires patience as well as tenacity.

Inspired by Mark Funk's Doe Lecture, where he analyzed word usage in *JMLA-*published articles to explore changes and trends within the profession, I looked to see how many times fake news, disinformation, and misinformation were used within JMLA [10]. The term fake news only showed up once. Elaine Martin's Doe Lecture discussed social justice and the role that medical librarians can play in a democratic society [11]. The term disinformation was in one article on the library's role countering infodemics regarding COVID-19 [12]. Misinformation retrieved twelve times, with it being used most often in article on MLA's InSight Initiative Summit. The audience discussed the absence of experts in public conversations of health-related topics and the responsibility of publishers and librarians to counter misinformation. The InSight Initiative Summit in 2018, stated “the growth of casual comment on social media on topics such as vaccination and lay people debating medical evidence is a huge concern,” and advocated for publishers and librarians to be more active in this area [13].

Jerry Perry in his Doe Lecture spoke about improving the quality of life through accurate health information. In 2019, he argued that each of us (medical librarians) are activists and need to own that role [14]. The problem with fake news is not that people fall for it but that it erodes trust in legitimate information. It can deepen ideological divides, disenfranchise people of their rights, and encourage violence. Fake news often impacts marginalized communities, people of color, and different ethnicities and religions. Since fake news impacts health, librarians combatting the spread of fake news through accurate health information are improving quality of life for others. Library collections, online tools, and collaborative educational opportunities are some ways for librarians to promote accurate health information and combat fake news.

Medical librarians must take ownership of their collections and purchase only scientifically valid resources. They must remove old or outdated materials as the science may be outdated For example, there are older books that refer to gender incongruence as a mental disorder. However, simply removing older editions is not enough, they must be aware of the fake medical information market and work to prevent those materials from infiltrating their collection. According OCLC World Cat and NLM Locator, books from Kevin Trudeau, an author who is a felon convicted of fraud for making misleading health claims in his books, can be found in medical libraries across the country, including the National Library of Medicine [15]. As champions of health information we need to be constantly aware and involved in our purchases, ask questions about the content and whether it promotes or obscures accurate health information.

There is more to the collection than books; librarians must also be aware of the thorny landscape of predatory journals. We can help prevent the spread of fake news through continued awareness and education on the complicated nature of predatory publishing. While the articles in predatory journals are not necessarily fake or wrong, it can be difficult to determine what is real given the lack of proper vetting of information in these journals.

Preprint articles, while helpful in breaking the barriers to getting important research out to the public to help speed up the treatments, can be fraught with inaccuracies that can cause more harm than good. A study in PLOS One found that nearly half of the preprint articles on COVID-19 found on medRxiv and later published in peer reviewed journals contained differences in data, title, and even conclusions. Those articles where the title changed made it difficult to track the article to verify the changes in information from the preprint version to the published peer review version [16].

In order to combat all this possible fake news, librarians should create, collect, and promote online tools on identifying and countering fake news to the people we serve. Library web pages listing tools like “Bad News” game or First Draft News can be helpful to students and medical professionals. In October 2022, the president of the American Medical Association discussed hearing from frustrated physicians working with patients whom they have seen for years and who have trusted their care, and these patients now make decisions against their medical advice based on fake news [17].

More medical librarians can reach out to other librarians to help prevent the spread of fake news. Public librarians have the experience of communicating and providing information to communities, and medical librarians have the experience of working with medical information. A public librarian and medical librarian team makes an effective consumer health information partnership. Academic medical librarians can partner with university departments already challenged by fake news. Journalism schools and schools of public health are two examples of natural partners to fight against fake news.

Artificial intelligence (AI) has added another complication and layer to the proliferation of fake news. AI is a tool. It is neither good nor bad; it is how people use AI and whether they use it properly as to how it impacts information. The answers that AI provides is related to the data available for it to use. Bad data coming in can equal bad answers going out. Since many AI programs use a variety of data available on the Internet, the data can be correct or questionable. The questionable data can be fraught with bias, misinformation, and outright lies, leading to potential ethical issues regarding the data and results. Librarians should be more engaged in the understanding of the data behind the AI to help ensure factual health information is provided.

Librarians can't be everything at all times, but we can use our strengths to address fake news within our libraries, institutions, and the profession as a whole. Librarians must get involved; simply relying on facts to do the work for us is not a solution. History has shown us there will always be fake news and there will always be new mediums and technologies by which it is spread. My favorite thing about being a medical librarian is finding information and sharing it with people, but we can't do that if fake news keeps getting in the way and muddying the message.

A lie may get halfway around the world before the truth has a chance to get its pants on. But we don't have to stand there and watch. Let's take it one leg at a time and get our pants on to help prevent it from spreading to the other half of the world.
